# Forging Hopeful Futures Community-Partnered Violence Prevention Intervention: Protocol for a Comparative Effectiveness Trial

**DOI:** 10.2196/90689

**Published:** 2026-04-29

**Authors:** Alison Journey Culyba, Julie Pulerwitz, Aniruddh Ajith, Sarah Barrett, Keona Blankenship, Alison Chin, Casey Diane Hanner, Christina Henry, Sarah Klotz, Naana Koranteng-Yorke, Kelsey Minter, Rikiyah Mixson, Deqa Mumin, Ashley Sabia, Emily Sierra, Nikole Saur, Tyia Wilson, Ann Gottert

**Affiliations:** 1 Department of Pediatrics, UPMC Children's Hospital of Pittsburgh, University of Pittsburgh, 120 Lytton Avenue, Pittsburgh, PA, 15213, United States, 1 4126926056, 1 4126928584; 2 Social and Behavioral Science Research, Population Council, Washington, DC, United States; 3 Department of Pediatrics, University of Pittsburgh, Pittsburgh, PA, United States

**Keywords:** violence prevention, youth, clinical trials, implementation science, positive youth development

## Abstract

**Background:**

Homicide is the third leading cause of death for US adolescents and the leading cause among Black youth. Youth violence also contributes to significant mental health burden and educational disruption, with the highest impact in neighborhoods with limited resources. Programs that address intersecting determinants, including social factors and limited economic opportunities, may reduce violence experiences and perpetration.

**Objective:**

This protocol paper describes a community-partnered, 2-arm cluster–randomized trial across 16 neighborhoods (12 in Pittsburgh, Pennsylvania, and 4 in the Washington, DC, region) evaluating the Forging Hopeful Futures (FHF) intervention, enrolling approximately 720 youth aged 13 to 19 years.

**Methods:**

FHF comprises 12 sessions delivered over 6 to 12 weeks by trusted community facilitators and addresses conflict resolution, peer and intimate partner relationships, youth leadership, and job readiness with connections to employment and mentorship. Comparison clusters receive enhanced usual care (individual wellness check-ins). Assessments occur at baseline, the end of the program, 3 months, and 6 months post-program. Primary outcomes are violence involvement (experience and perpetration); secondary outcomes include experiences of multiple types of violence (eg, relationship abuse, sexual violence, bullying, and weapon carrying). Implementation data are collected using RE-AIM (reach, effectiveness, adoption, implementation, and maintenance)–informed tools and qualitative interviews.

**Results:**

The study was funded in October 2022, and recruitment began in July 2023. As of December 1, 2025, the study had enrolled 542 participants, with follow-up expected to continue through July 30, 2026. Data analysis for primary end points is expected on January 1, 2027. Primary analyses will estimate intervention effects on recent violence perpetration using generalized linear mixed models with random effects for neighborhood and participant, adjusting for baseline values and city. Exploratory analyses will examine mediation (eg, shifts in attitudes) and moderation (eg, baseline risk profiles).

**Conclusions:**

This trial is designed to provide rigorous effectiveness and implementation evidence to inform policy and practice in youth violence prevention. If demonstrated to be effective, FHF could serve as an integrated, scalable model that addresses the social and economic drivers of youth violence and leverages community partnerships for sustainability.

## Introduction

### Background

Youth violence is a pervasive public health problem in the United States, with serious and lasting consequences for physical and mental health [1-4]. Homicide, the most severe consequence of interpersonal violence, remains the third leading cause of death among US adolescents aged 15 to 19 years and the leading cause of death among Black youth in this age group [5,6]. Firearms became the leading cause of death for adolescents aged 14 to 19 years as of 2016, and in 2023, rates for Black adolescents remained far higher than those of other races [7]. Adolescents in high-poverty areas face markedly higher exposure, with homicide rates more than four times higher in high-poverty census tracts than in low-poverty areas [8].

Population-based surveys also document high levels of nonfatal violence and weapon-related risk among high-school students [9]. The National Youth Risk Behavior Surveillance System data show that in 2021, 13% of high school students reported being in a physical fight in the past year, 5% reported carrying a weapon in the past 30 days, and 8% had been threatened or injured with a weapon on school property [10,11]. The 2023 National Youth Risk Behavior Surveillance System provides the most current national estimates, showing that nearly 1 in 8 students experienced at least one form of physical violence in the past year [12].

Youth violence also has lasting developmental and societal consequences, such as impacts on educational attainment [13] and community well-being [14]. Exposure to violence, whether as a victim, perpetrator, or witness, is associated with 2- to 3-fold higher odds of depression and posttraumatic stress disorder [15], increased suicidal ideation [4], and an elevated risk for substance misuse [16]. Longitudinal analyses confirm that cumulative exposure to violence in adolescence predicts an elevated risk for firearm carrying, aggression, and mental health difficulties into early adulthood [17,18]. At the community level, persistent violence has a huge economic cost, erodes perceptions of safety, reduces civic engagement, and perpetuates cycles of social disinvestment [19,20]. Approaches to reduce violence focused on high-school-age youth are urgently needed.

### Prior Work

Evidence from recent population studies [4,5] shows that experiences of multiple forms of violence often co-occur, with cumulative impacts on mental health and behavioral risk. Our work has demonstrated links between victimization and perpetration across various types of violence (eg, peer violence, weapon violence, sexual or relationship violence, bullying, and homophobic teasing) among youth [21].

Youth violence is a complex issue driven by a combination of individual, family, community, and societal factors [22,23]. Individual risk factors include a history of violence, substance abuse, and emotional distress. Family factors, such as mental illness and poverty, can also increase the likelihood of youth violence. Community- and societal-level factors like lack of opportunity, exposure to violence, harmful gender norms, and racial discrimination can contribute to a youth’s involvement in violence. Discrimination contributes both directly to psychological distress and indirectly to violence through mechanisms such as school exclusion and diminished economic opportunity [24-26]. Among young men, restrictive conceptions of masculinity and gender norms, such as the expectation that men “never show weakness,” are associated with significantly higher odds of aggression, bullying, and dating violence [27,28]. Understanding how individual-level risk factors intersect with neighborhood contexts is essential for identifying leverage points for intervention.

At the same time, protective assets can buffer the translation of adversity into violence. Future orientation, a belief in one’s ability to achieve meaningful goals, and supportive relationships with adults and peers have been linked to lower odds of multiple violence outcomes [29,30]. Collective efficacy within neighborhoods can mitigate the impact of exposure to life-threatening violence on youth development [31]. Creating opportunities for youth leadership can function as protective mechanisms, reducing the translation of adversity into violence while building community resilience [32]. Evidence from the United States and abroad indicates that programs explicitly questioning and shifting restrictive views toward men’s and women’s roles and responsibilities—or inequitable social and gender norms—can reduce sexual and relationship violence and HIV risk [33,34]. When paired with employment readiness and opportunity pathways, these programs have the potential to reduce other forms of violence as well [1,34]. These insights point toward multicomponent strategies that simultaneously address multiple drivers and protective assets to reduce violence.

Intervention research is increasingly aligned with this synthesis. National recommendations for preventing youth violence emphasize coordinated strategies that create protective environments, strengthen youth skills, connect youth to caring adults and activities, and support economic opportunities [35]. Results from a randomized controlled trial (RCT) over a decade ago found that engaging youth in high-violence environments in summer programs significantly reduced violence [36]. A recent multisite longitudinal study found that neighborhoods with sustained investment in youth programs saw 18% lower rates of violent offending over a 5-year period, underscoring the potential for upstream prevention [37]. Synergistic models that address cross-cutting risk and protective factors may, therefore, offer a scalable approach to reducing multiple forms of violence simultaneously.

In our prior work, a community-based program promoting a positive and caring sense of manhood for adolescent boys and young men—called “Manhood 2.0”—and a related job readiness comparison, both reduced sexual violence perpetration [34]. This suggests promise for cross-cutting reductions in youth violence when gender, racial, and economic drivers are addressed together [38,39]. Other job-readiness and mentoring programs have also shown promise for improving prosocial engagement among youth in environments of high violence exposure, though effects on violence outcomes have been heterogeneous, again suggesting the value of an integrated model that addresses multiple drivers simultaneously [35].

At this point, significant gaps remain in addressing youth violence. Few rigorously evaluated US programs intentionally integrate content that simultaneously addresses multiple drivers of youth violence. Moreover, relatively little research interrogates mechanisms of change (eg, whether shifts in attitudes, gains in future orientation, and increased job skills explain reductions in violence involvement) or examines moderators such as prior exposure to violence or structural disadvantage. A pragmatic, community-delivered intervention evaluated through a cluster-randomized design can address these gaps while producing evidence relevant for scale and sustainability. In short, youth violence remains a pressing public health priority, and rigorous evaluation of promising, theory-driven, and pragmatic approaches is necessary to determine whether such an integrated model reduces violence and to identify pathways of effect.

### Study Objectives

This protocol describes the rationale and design for a 3-year, 2-arm, community-partnered cluster–randomized trial of *Forging Hopeful Futures* (FHF), an intervention that integrates curriculum content on violence prevention and conflict resolution, critical-consciousness building around key social drivers, youth leadership, and job-readiness with facilitated connections to employment and mentoring. The trial enrolls high school adolescents (aged 14‐19 y) in neighborhoods with limited resources and high violence exposure in Pittsburgh, Pennsylvania, and the Washington, DC, region. Guided by social determinants of health theories and implementation science frameworks, the study has 4 objectives. First, to assess whether youth in neighborhoods assigned to FHF, relative to enhanced usual programming, exhibit greater reductions in violence involvement (experience and perpetration). Second, to examine whether changes in key secondary outcomes—such as mental health, gender attitudes, and improvements in future orientation—mediate and help explain intervention effects on violence outcomes. Third, to evaluate moderation of intervention effects by baseline profiles, including prior violence exposure and neighborhood disadvantage, thereby identifying for whom FHF is most effective. Fourth, to document implementation processes, feasibility, and fidelity, informing future scale-up and adaptation.

Taken together, the study is designed to advance evidence for preventing youth violence and promoting healthy development among adolescents living in communities with limited resources. By addressing the intertwined influences of relationship dynamics, community violence, and economic challenges, this study aims to advance the science of youth violence prevention and inform policies and programs that promote safety and thriving for marginalized adolescents.

## Methods

### Study Design and Setting

**Figure 1.**
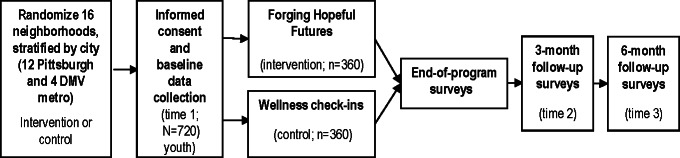
Two-arm cluster–randomized controlled trial study flow. DMV: Washington, DC, and Maryland metro areas.

This is a community-partnered, 2-arm cluster-RCT to evaluate FHF in 16 neighborhoods (clusters) across Pittsburgh, Pennsylvania, and the Washington, DC, metro area (Figure 1). Neighborhoods are the unit of randomization to minimize contamination and align with community delivery. Approximately 45 youth (aged 13‐19 y) will be enrolled per neighborhood (target N≈720). Assessments occur at baseline (T1), end-of-program (6‐12 wk after baseline), 3 months (T2), and 6 months (T3) postprogram. A mixed methods design integrates quantitative surveys with qualitative interviews and implementation tracking aligned with the RE-AIM (reach, effectiveness, adoption, implementation, and maintenance) framework, enabling rigorous effectiveness testing alongside implementation evaluation.

### Participants and Recruitment

Eligible participants are adolescents aged 13‐19 years who live, attend schools, or use participating facilities in a randomized neighborhood, can complete study procedures in English, and can provide follow-up contact information. Youth will be identified through community-based organizations, outreach by neighborhood facilitators, peer referrals, and partnerships with local health, social service, and youth development programs. After eligibility screening, informed consent or assent will be obtained in private settings. The study was approved by the University of Pittsburgh with a waiver of parental permission (for youth aged 13‐17 years) and a waiver of written documentation of consent.

### Randomization and Allocation Concealment

Th*e* trial design builds on primary prevention principles that emphasize a comprehensive, theory-driven approach, sufficient dosage (through repeated exposure to content), and youth participation balanced with the feasibility and cost of implementation approaches. Randomization is performed at the neighborhood level (ie, cluster) to reduce the risk of contamination. Neighborhoods are stratified by city (Pittsburgh vs Washington, DC, and Maryland metro areas [DMV]) and randomized 1:1 to FHF or enhanced usual care (wellness check-ins) by the study statistician using reproducible code. Randomization lists are generated prior to enrollment and implemented centrally; site teams will be notified of the assignment after cluster confirmation. Analyses will be conducted with masked arm labels, and outcome assessments will use standardized instruments to minimize bias.

### Intervention and Comparison Arms

**Figure 2.**
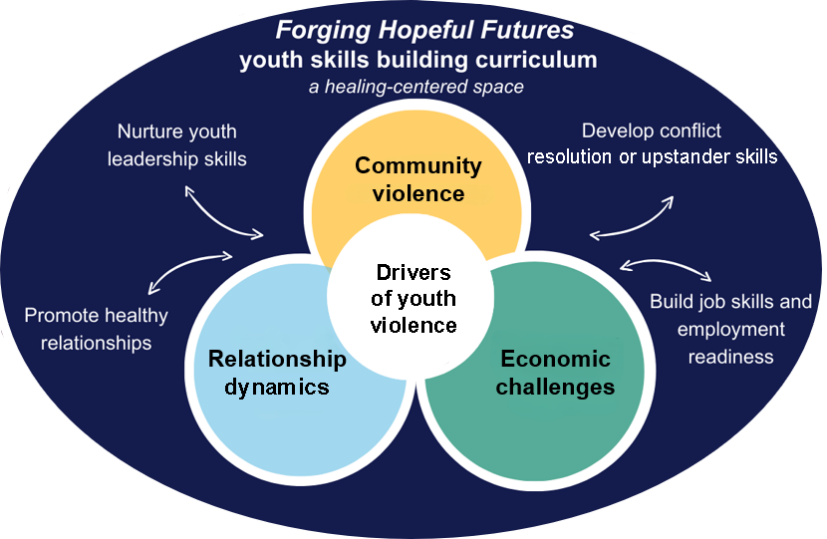
Conceptual model underlying Forging Hopeful Futures.

FHF comprises 12 facilitated sessions (≈2 h each) delivered over 6 to 12 weeks in community settings by trained local facilitators. The program uses strength-based and healing-centered approaches to nurture youth leadership skills, promote healthy relationships, develop conflict resolution and upstander skills, and build job skills and employment readiness (Figure 2). Facilitators receive standardized training, ongoing coaching, and fidelity monitoring using session checklists and observation rubrics. Near program completion, youth meet workforce partners and mentors to support concrete next steps.

Guided by our previous work demonstrating the violence prevention effects of both skills-building curricula and job readiness training [34], FHF was developed to explore the impact of combining this content into a unified intervention. The FHF curriculum was adapted by the study team by integrating content from four existing curricula: Manhood 2.0 [40], Sisterhood 2.0 [41], Creating Peace [42], and job skills training [43]; new synergistic content was also added. Community partners from youth-serving organizations worked with the FHF investigative team to synergize these evidence-based interventions into the FHF curriculum (Table 1). Community facilitators helped develop the facilitator and youth curriculum guides. We pilot-tested the FHF curriculum in one neighborhood not involved in the RCT and made additional refinements.

**Table 1.** Forging Hopeful Futures curriculum content.

Session topic	Curriculum sources	Session content
1. Exploring identity	Manhood 2.0, Sisterhood 2.0, and Creating Peace	Set goals, examine community norms, promote knowledge, and positive identity.
2. Understanding our emotions and experiences	Manhood 2.0, Sisterhood 2.0, and Creating Peace	Share experiences of violence, posttraumatic growth, and vicarious resilience.
3. Healthy relationships	Manhood 2.0, Sisterhood 2.0, and Creating Peace	Support discussions of healthy relationships, consent, and signs of abuse through interactive dialogue and scenarios
4. Examining history and power	Creating Peace	Explore how power affects interpersonal relationships, community violence, and building strategies to respond
5. Building conflict resolution or upstander skills	Manhood 2.0, Sisterhood 2.0, and Creating Peace	Develop nonviolent communication strategies to manage interpersonal conflict and safely intervene using upstander behaviors
6. Strengthening job skills	Job skills training	Develop workforce skills to promote job readiness: identifying strengths and skills, resumes, letters of reference, and finding employment opportunities
7. Succeeding in the job search	Job skills training	Develop workforce skills to promote job readiness (part II): job applications, interviews, and preparing for work
8. Empowering youth as change agents	Creating Peace	Define and build leadership skills through an empowerment framework, become a community leader, introduce photovoice as a catalyst for change
9. Envisioning a collective future	Creating Peace	Create a collective vision for a safe and supportive community using human-centered design activities
10. Networking for job success	Job skills training	Meet with job readiness programs and community mentoring programs and work with facilitators to identify and connect with employment, mentorship, and leadership opportunities
11. Reflecting and preparing to share	New synergistic content	Preparing presentations, practicing leadership skills, and showcasing visual and written expressions of empowerment
12. Sharing and celebrating with community	New synergistic content	Youth present their project celebrating identity, strengths, and skills to the broader community; graduation from program with community leaders, adult allies, and workforce development program leaders present

Youth in the comparison arm receive an individual wellness check-in (in-person or by phone) with tailored referrals to community resources and optional follow-up. This occurs through a strengths-based conversation focused on wellness resources, conducted by trained community facilitators and staff, typically lasting 20 minutes. Youth are offered the option of additional phone check-ins to coordinate access to community resources and connection to individualized behavioral health supports, if desired.

### Measures

The primary outcome of interest is the change in violence perpetration over the past 3 months, assessed via a self-report composite measure of physical fighting, threatening someone with a weapon, and injuring someone with a weapon at T2 (Table 2). Secondary outcomes focus on violence perpetration (as per the primary outcome measure) measured at 6 months (T3), as well as the change in self-reported use of a range of types of violence in the past 3 months, including relationship abuse, sexual violence, bullying, weapon carrying, homophobic teasing, and sexual harassment.

**Table 2.** Primary and secondary outcome measures.

Outcomes	Measures description	Time frame
Primary outcome		
Change in recent violence perpetration at 3 months	Self-report on 3 items adapted from the Youth Risk Behavior Surveillance Survey, using a past-3 month reporting interval: (1) physical fighting (“How many times were you in a physical fight?“), (2) threatening someone with a weapon (“How many times have you threatened someone with a weapon such as a gun, knife, or club?“), and (3) injuring someone with a weapon (“How many times have you injured someone with a weapon such as a gun, knife, or club?"). Each item will be assessed with 8 frequency response categories from 0 to 12 or more times. A summary score will capture the past 3-month incidence of all 3 behaviors (possible range: 0‐21; lower score indicates better outcome)	At baseline and 3 months after program conclusion
Secondary outcomes		
Change in recent violence perpetration at 6 months	This will be measured using the same 3 items outlined in the primary outcome, but assessed as a change from baseline to 6 months after program conclusion. This includes self-report on 3 items adapted from the Youth Risk Behavior Surveillance Survey, using a past-3 month reporting interval: (1) physical fighting (“How many times were you in a physical fight?“), (2) threatening someone with a weapon (“How many times have you threatened someone with a weapon such as a gun, knife, or club?“), and (3) injuring someone with a weapon (“How many times have you injured someone with a weapon such as a gun, knife, or club?"). Each item will be assessed with 8 frequency response categories from 0 to 12 or more times. A summary score will capture the past 3-month incidence of all 3 behaviors (possible range: 0‐21; lower score indicates better outcome)	At baseline and 6 months after program conclusion
Change in relationship abuse at 3 months	Ten abusive behavior items, modified from Conflict Tactics Scale-2, assess for perpetration against dating partners or toward peers. Summary score of any recent use of violence in the past 3 months (physical, sexual, and emotional relationship abuse) calculated as one point for each behavior then summed (possible range: 0‐10; lower score indicates better outcome).	At baseline and 3 months after program conclusion
Change in sexual violence perpetration at 3 months	Four sexual violence perpetration items assess recent sexual violence perpetration (any or none). Summary score of any recent sexual violence perpetration in the past 3 months, calculated as 1 point for each behavior then summed (possible range: 0‐4; lower score indicates better outcome).	At baseline and 3 months after program conclusion
Change in cyber dating abuse and peer abuse	Eight items assess using technology to perpetrate abuse against a dating partner or peers. Summary score of any recent use of technology to perpetrate abuse against a dating partner or peers in the past 3 months, calculated as 1 point for each behavior then summed (possible range: 0‐8; lower score indicates better outcome).	At baseline and 3 months after program conclusion
Change in bullying perpetration	Three items to indicate frequency of bullying perpetration on 3-point frequency scale. Mean score of any recent bullying perpetration in the past 3 months, calculated as a mean score across the 3 items (possible range: 0‐2; lower score indicates better outcome).	At baseline and 3 months after program conclusion
Change in weapon carrying	One item adapted from the Youth Risk Behavior Surveillance Survey asking about frequency of carrying a weapon in the past 3 months (measured on a 5-point frequency scale from 0 days to 6 or more days; possible range: 0‐4; lower score indicates better outcome).	At baseline and 3 months after program conclusion
Change in homophobic teasing	Five-item scale with participants reporting how often they have perpetrated listed behaviors in the past 3 months (each item measured on a 5-point frequency scale from 0 to 7 or more times). Mean score of recent homophobic teasing, calculated as a mean across the 5 items (possible range: 0‐4; lower score indicates better outcome).	At baseline and 3 months after program conclusion
Change in sexual harassment	Five items assess frequency of perpetrating sexual harassing behaviors in the past 3 months, assessing perpetration frequency on a 4-point frequency scale. Mean score of recent sexual harassment perpetration in the past 3 months, calculated as a mean across the 5 items (possible range: 0‐3; lower score indicated better outcome).	At baseline and 3 months after program conclusion

**Figure 3.**
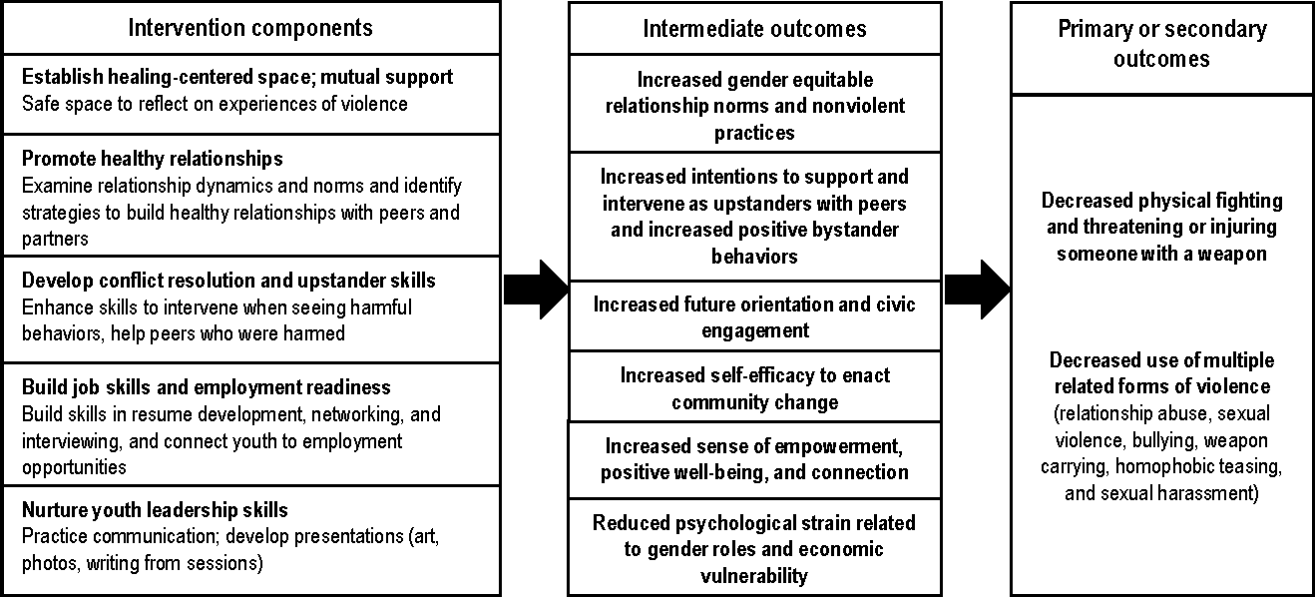
Forging Hopeful Futures intervention components and hypothesized outcomes.

Intermediate outcomes will assess violence-related attitudes and behaviors, as well as youth empowerment, future orientation, civic engagement, self-efficacy, and other key social variables (eg, gender attitudes) [44] (Figure 3). Prior violence exposure (witnessing and victimization) and neighborhood context will be assessed at T1. Demographics include age, gender, race or ethnicity, and housing or education context.

### Data Collection and Procedures

Surveys are administered via secure tablets or web links in private spaces at each time point (T1–T3). To protect confidentiality for sensitive topics, surveys are anonymous and linked across time using a self-generated code derived from stable personal prompts [45]. Research staff provide brief instructions and remain available if technical or literacy support is needed. To enhance retention, the team uses multimodal reminders, flexible scheduling, and community partner touchpoints; incentives are modest and escalate across follow-ups. A subset of FHF youth and facilitators participates in qualitative interviews to contextualize implementation facilitators and barriers.

### Sample Size and Power

In determining the number of neighborhood clusters to include in the study, we sought to balance expected effect sizes with feasibility and budgetary constraints for a 3-year multicity study. Our selection of 16 clusters was informed by a previous cluster-RCT led by our team to evaluate the Manhood 2.0 curriculum and a pilot feasibility study of the Sisterhood 2.0 program (NCT02427061 and NCT04388696, respectively). We used these study findings to guide assumptions for our sample size calculations [34,39,40,46]. Sample sizes for each outcome were based on clinically meaningful differences between treatment groups with respect to changes in outcomes across time (ie, intervention effect). For the primary outcome of self-reported youth violence perpetration (fighting or weapon violence perpetration) in objective 1, the necessary sample size was calculated based on traditional methods that assume a fixed number of clusters. If we assume 8 neighborhoods randomized per arm with a conservative within-site correlation of 0.01, and 20% attrition at 3 months, we need 360 participants per arm (total N=720) to detect a standardized effect size of 0.39 with 80% power. This translates to a 35% relative reduction in the primary outcome, assuming a 57% rate in the control arm at 3 months. For all secondary outcomes, we have similar power to detect a standardized effect size of 0.39.

### Statistical Analysis

Descriptive statistics will be used to summarize the sample with regard to baseline characteristics of interest. Means and SDs will be presented for continuous variables, while sample proportions will be provided for categorical variables.

Primary assessment of intervention effects will be based on intent-to-treat analyses. As-treated effect parameters will be estimated in secondary, exploratory analyses. We will conduct intervention intensity-adjusted analyses (estimating the dose that participants received) by substituting a score between 0 and 1 for the treatment group. Additionally, we will conduct per-protocol analyses, with participants exposed to “intervention as intended” in the treatment arm and all others treated as controls (ie, 0). Generalized linear mixed models will be used to account for stratification by city, correlation between youth from the same neighborhood, as well as the correlation between longitudinal observations from the same youth. SAS (Statistical Analysis System) software (SAS Institute) will be used for all statistical analyses. All hypotheses will be 2-sided tests with a significance level of 5%, and 95% CIs will accompany all sample statistics.

Participation bias will be assessed by comparing the age and race or ethnicity of youth participating in the study to each neighborhood’s overall demographics of youth (based on census tract data). Attrition analysis will be conducted by comparing youth who completed follow-up surveys with those who did not, based on demographics as well as outcomes measured at baseline. Mechanisms for missing data will be investigated by comparing important covariates between youth with and without missing data at each time point. We will characterize these mechanisms as (1) missing completely at random, (2) missing at random, or (3) not missing at random. If the nature of our missing data is ignorable (either missing completely at random or missing at random), we will use imputation methods, such as multiple imputation via chained equations, to handle dropout. Ultimately, sensitivity analyses will be conducted to compare the results of our imputation methods to complete-case and available-data analyses.

Exploratory mediation of intervention effects will be tested using methods such as structural equation modeling, and moderation will be examined using methods such as latent class analysis. Preplanned subgroup analyses will explore heterogeneity by sex, prior violence exposure, and neighborhood context.

### Ethical Considerations

The protocol for this study has been approved by the University of Pittsburgh Institutional Review Board and has been registered on ClinicalTrials.gov (NCT05743478). Procedures prioritize confidentiality and minimize risk: surveys are anonymous; distress protocols include on-site support, warm handoffs to behavioral health services, and mandatory reporting limited to legally required circumstances. Parental permission waivers and oral assent procedures for youth will be used where justified to reduce participation barriers. All staff are trained in trauma-informed, youth-centered practices. During the recruitment process, all potentially eligible youth will be reminded that participation is completely voluntary and that whether or not they participate will have no effect on their participation in any of the organization’s programs and activities. Participants can choose not to answer any questions or discuss any topics that make them uncomfortable. All data collection materials will be anonymous and will contain no personal identifying information. Data are further protected by a Certificate of Confidentiality from the Centers for Disease Control and Prevention (CDC). Following surveys and interviews, we will briefly check in with each participant to assess for emotional distress. Should a participant display signs of emotional distress or disclose to the research assistant that they are in danger of hurting themselves, hurting others, or being hurt, the research assistant will follow an established protocol to notify appropriate community agency personnel and ensure that the participant receives urgent evaluation. Additionally, a systematic review of notes from research assistants will be conducted to ensure that participants experiencing distress are being connected directly with the community site leads or administrators, receiving educational materials, and being referred appropriately; this includes ensuring that all research assistants document asking each participant about emotional distress after completion of the survey and interview and giving all participants a set of resources to take with them after completing the survey. If we learn that a participant is in danger of hurting themselves, hurting others, or being hurt, we will inform the appropriate authorities (eg, Children, Youth, and Families), as we are required to do under Pennsylvania law. Youth will receive US $20 for completing the baseline survey, US $30 for the end-of-program survey, US $30 for the 3-month follow-up survey, US $50 for the 6-month follow-up survey, and US $30 for participating in a semistructured interview.

A data safety plan documents adverse event monitoring and reporting. Given the sensitivity of the questions being asked regarding violence perpetration, we also established an internal data safety and monitoring plan. The senior research coordinator is responsible for daily monitoring of data and safety. They work with research assistants to ensure that all data are collected and stored securely. The research team, consisting of the principal investigator (AJC), research coordinator, research assistants, data analysts, and other research staff as appropriate, meets weekly to review study progress, the status of data collection, and the safety of participants. Concerns will be reported to the institutional review board in accordance with institutional and CDC requirements.

## Results

This comparative effectiveness study was funded by the CDC in October 2022. Study recruitment began in July 2023 and data collection began on July 10, 2023. As of December 1, 2025, the study has enrolled 542 participants, with follow-up expected to continue through July 30, 2026. Data analysis for primary end points is expected on January 1, 2027. Feasibility and implementation metrics (eg, recruitment rates, session attendance, fidelity scores, and acceptability from youth or facilitators) will be summarized. Primary and secondary outcomes will be analyzed after the final follow-up window closes, with planned CONSORT (Consolidated Standards of Reporting Trials) flow, baseline characteristics, and effect estimates reported per trial registration. Baseline data analysis is underway and we expect to publish the primary trial results by spring 2027.

## Discussion

### Expected Findings

This protocol advances a multilevel approach to youth violence prevention that integrates skill-building content and economic opportunity within a community delivery model. The cluster design aligns with real-world implementation, while mixed methods support interpretation and scalability. Potential risks include attrition and variable neighborhood capacity; mitigation strategies include strong community partnerships, flexible scheduling, transportation supports, and continuous facilitator coaching. If effective, FHF could address persistent youth violence by targeting modifiable drivers across the social ecology.

Local data underscore the acute burden in study settings. In Allegheny County, Pittsburgh, homicide rates for Black males aged 15 to 24 years have remained 50 times higher than the US average for all youth, with more than 67% of youth homicides being firearm-related [47]. In 2021, Allegheny County recorded its highest rate of firearm-related youth fatalities in over a decade [47]. In Washington, DC, firearm-related youth deaths increased by over 85% from 2011 to 2020, and while 2024 data show modest declines, rates remain far above national averages [48].

This study has several potential limitations. Sample size calculations were based on prior related studies, and it is possible that correlations and effect sizes may differ in this trial, which could impact the power to detect anticipated effects. The violence perpetration behaviors are self-reported, leaving them subject to potential biases and inaccuracies—a known challenge in adolescent violence research [49-56]. In spite of these limitations, self-reported data are widely used to assess youth violence in epidemiologic and intervention research [12]. The self-generated anonymous code and computerized survey are intended to enhance accurate reporting of sensitive items; youth are reminded to be as honest as they can. Many behaviors youth report witnessing or perpetrating are not readily available through administrative data [57-59]. While every effort will be made to prevent intervention and control sites from mixing, it is possible that facilitators or youth exposed to FHF may contribute to some contamination in control sites by participating in youth activities at a control site. Any cross-over among participants will be documented as a potential threat to validity. As the primary analysis involves intention-to-treat using participant-level violence outcomes, movement to a new neighborhood is unlikely to measurably impact findings. We are attempting to balance the goals of community collaborators to offer an intervention to all youth with the budgetary and personnel limitations inherent in the 3-year study timeframe. An attention control design with 12 sessions of an alternative program would be cost-prohibitive. Although “true” controls may be considered optimal experimental design, when working in communities with concentrated disadvantage, our research team has found that offering some intervention in comparison neighborhoods is critical for recruitment and retention. Comparing FHF participants to youth receiving this enhanced usual care offers a pragmatic effectiveness trial. Expansion of FHF to DMV may require adaptations to implementation processes and programming. Our focus on implementation processes through mixed methods data collection across all stages of implementation, coupled with regular meetings with key personnel in Pittsburgh and DMV, is designed to identify and address barriers in real-time.

### Conclusions

This community-partnered cluster–randomized trial of FHF, a violence prevention intervention addressing the intertwined influences of relationship dynamics, community violence, and economic challenges, is designed to generate actionable evidence for a scalable youth violence prevention strategy. The findings will inform community programs and policy investments that promote safer, healthier developmental contexts, including for adolescents in neighborhoods with limited resources.

## Supplementary material

10.2196/90689 Peer Review Report 1 Peer review report by: ZCE1 AW (4B) - National Center for Injury Prevention Special Emphasis Panel CE22-005: Research Grants for Preventing Violence and Violence Related Injury, National Center for Injury Prevention and Control (National Institutes of Health, USA).

## Abbreviations

CDC: Centers for Disease Control and Prevention
CONSORT: Consolidated Standards of Reporting Trials
DMV: Washington, DC, and Maryland metro areas
FHF: Forging Hopeful Futures
RCT: randomized controlled trial
RE-AIM: reach, effectiveness, adoption, implementation, and maintenance
SAS: Statistical Analysis System
