# Supplementary material for: Forging Hopeful Futures Community-Partnered Violence Prevention Intervention: Protocol for a Comparative Effectiveness Trial
Source: JMIR Res Protoc. 2026 Apr 29;15:e90689. doi: 10.2196/90689 (PMC13128155; doi:10.2196/90689)
Supplement: Peer Review Report 1 [file resprot-v15-e90689-s001.pdf]

SUMMARY STATEMENT
(Privileged Communication)

PROGRAM CONTACT:
Amanda Garcia-Williams
(770) 488-3936
gvl8@cdc.gov

Release Date: **08/12/2022**
Revised Date:

Application Number: **1 R01 CE003502-01**

Principal Investigator

CULYBA, ALISON J.

Applicant Organization: UNIVERSITY OF PITTSBURGH AT PITTSBURGH

Review Group: **ZCE1 AW (4B)**
National Center for Injury Prevention Special Emphasis Panel
CE22-005: Research Grants for Preventing Violence and Violence Related Injury (R01)

Meeting Date: **05/10/2022**
RFA/PA: **CE22-005**
Council: **OCT 2022**
Requested Start: **09/30/2022**

Project Title: **Forging Hopeful Futures: A Racial and Gender-Justice Program to Reduce Youth Violence**
SRG Action: **Impact Score:30**

Human Subjects: **30-Human subjects involved - Certified, no SRG concerns**
Animal Subjects: **10-No live vertebrate animals involved for competing appl.**
Gender: **1A-Both genders, scientifically acceptable**
Minority: **1A-Minorities and non-minorities, scientifically acceptable**
Age: **6A-Children and Adults, scientifically acceptable**
NIH Defined Phase III Clinical trial

Project Year	Direct Costs Requested	Estimated Total Cost
1	294,907	401,894
2	294,063	400,743
3	291,470	397,210
TOTAL	880,440	1,199,847

1R01CE003502-01 Culyba, Alison

BUDGET MODIFICATIONS ARE RECOMMENDED

RESUME AND SUMMARY OF DISCUSSION: This application, by a seasoned Principal Investigator (PI), proposes to refine and evaluate a youth empowerment intervention, Forging Hopeful Futures (FHF), extending previous work by combining economic justice content from job readiness training, racial and gender justice content from gender-transformative programming, and leadership building as a novel multi-level violence prevention intervention. The specific aims are to: 1) evaluate the effectiveness of the FHF intervention compared to wellness check-ins (control) among 14- to 19-year-old youth to decrease self-reported use of physical fighting and threat/injury with a weapon (primary outcome) and multiple related forms of violence (i.e., physical, sexual, and emotional relationship abuse, sexual violence, bullying); 2) examine mechanisms through which the intervention creates impact and how pre-intervention risk and protective factors moderate intervention effects on multiple forms of violence; and 3) examine implementation processes that facilitate intervention acceptability, reach, integration, fidelity, and engagement as well as costs associated with intervention implementation across neighborhoods. Reviewers determined this proposal to have high potential for public health impact.

Regarding significance, the application addresses an important problem and is poised to make a significant impact on the field by attending to intersectionality. The results of the project have high potential for generating information related to racial and ethnic disparities, which is pertinent to the Notice of Funding Opportunity (NOFO). The proposed study is an excellent fit to the NOFO, given its focus on prevention of youth violence (YV). The study will involve 720 youth from eight neighborhoods that will be randomly assigned to receive the intervention and eight neighborhoods assigned to a control condition involving wellness check-ups. The groundwork for the proposed project has been laid through the team's strong collaborative partnerships with the community agencies that will be engaged in recruiting participants and carrying out the intervention. The range of forms of violence that will be addressed is impressive, including gender-based violence, homophobic teasing, bullying, sexual harassment, and sexual violence and coercion. The study is positioned to make a significant impact on the participants and communities in which the intervention will be implemented. In the short- and long-term, the project is designed to meet the needs of communities. For instance, the workforce development opportunities that are built into the intervention and the research participation process have the potential to directly address economic disparities among the participants. In terms of the investigators, this is a highly accomplished team that has expertise and considerable experience in conducting intervention research with the communities that will be the focus of the proposed project. The investigators have laid considerable groundwork for the project, including conducting previous research that has used many of the facets of what they are putting together in the novel FHF intervention. The investigators have established strong collaborative partnerships with community agencies that are necessary for the community empowerment and community participation perspectives that inform their approach to research. The roles and responsibilities of each member of the research team are clearly described. The Population Council has decades of experience and expertise in developing innovative methods to measure gender norms. While much of that has been in international settings, the proposed study would adapt those methods to the United States (US) setting. There has been some translation in US settings that the application notes. The expansion to the District of Columbia (DC) and the DMV (DC, Maryland, Virginia) area is a strength in terms of improving the generalizability of the findings. Regarding innovation, the proposed study is highly novel in the way in which it addresses the intersectionality of race, ethnicity, gender, and economic inequities in a way that is unique, and it is also drawn through a very well-articulated conceptual model. The community participatory and community empowerment aspects of the methods are also highly innovative, including the engagement of the Youth Advisory Committee (YAC) and involving community partners in the design of the intervention and surveys. The examination of youth profiles through latent class analysis

(LCA) is innovative and has the potential to make important contributions to the understanding of intersectionality of risk factors and could be useful in moving toward a more precision medicine approach in which the effectiveness of interventions is increased by tailoring and individualizing them. The focus on delivering the intervention outside of the school setting is innovative and well-reasoned, given that many at-risk youth are not spending their time in school. In terms of the approach, the proposed study builds on the Pittsburgh study. The inclusion of multiple sites is a strength. The conceptual model underlying the research questions is very clear and well-articulated. The cluster randomization trial (CRT) design stratifies both by site and by prior intervention arm. The control group is clearly described and well defined. The outcome measures are clearly identified and well operationalized. The data to be collected are appropriate to the research questions. The data analytic plan is appropriate and methodologically rigorous. Not only will outcomes be evaluated, but also the underlying conceptual model will be tested as the purported mechanisms by which those outcomes are achieved. Considerable thought has been given to how to make the intervention more responsive and acceptable to the community, including by designing a control group that is offered something rather than nothing. The wellness check-ins are a good alternative to just telling people that they would be languishing while awaiting the intervention, which is not received very well by communities that are lacking in resources. A good job is done in attending to the principles of implementation science, including ongoing assessment of fidelity of the intervention through multiple methods. Several potential challenges and possible alternative strategies are clearly outlined. The application clearly describes the necessary environment and institutional supports. There are strong existing partnerships among the investigators, their institution, and the community agencies needed to do the proposed work.

Weaknesses also were identified. Regarding significance, it is mildly disappointing that while the application takes a racial, gender, and economic equity lens, all of the interventions are very individual-focused. This is more like an inoculation resilience-building type of strategy, which is not a structural intervention. While there is nothing wrong helping individuals fare better in their environments, it is pitched as a structural intervention even though it is not targeting structural factors. The Phenomenological Variant of Ecological Systems Theory (PVEST) theoretical framework is brought up at the end of the significance section, but it is never clearly explained or incorporated. The use of PVEST and the expansion of that theoretical framework for implementation of the FHF intervention, especially with minority youth, would be a significant strength to the study. With respect to the investigators, the level of effort for some of the team members seems low. The level is as low as 5% for some team members, which seems inadequate based on the scope of the study. There is a minor weakness with regard to innovation in that the team missed out on opportunities to take a more innovative holistic approach by examining factors at the community and social levels that also could act as mechanisms of change in this intervention. The application notes the use of LCA as an innovation, but then points to a study that used LCA for the same topic area. Concerning the approach, it does not appear that any piloting has been done of the racism or discrimination components. This raises a concern about the potential for iatrogenic effects related to utilizing that sensitive type of work with no pilot data to support it and without strong theoretical data for how this approach actually would help as opposed to making things worse. It appears that only perpetration and not victimization would be assessed, which seems like a missed opportunity. Table 2 introduces a number of terms and concepts that are not initially defined. Therefore, it seems like the curriculum is based off of potential factors that are not laid out clearly in the significance section. Examples include strengths-based and healing-centered approach. Some reviewers felt that the participant recruitment strategies represent one of the least well-explicated elements of the research design. Two strategies are mentioned, one of which is referrals from community agencies and the other is snowball recruitment in which participating youth will be requested to refer their peers. The concern is that the community agencies might be directing youth who already have been involved in violence to the intervention, which might not meet the goals of prevention as well as other recruitment strategies and could skew the sample. The concern with snowball recruitment is that it might result in a convenience sample with a limited range of characteristics in that youth are likely to recruit other youth who are much like themselves from their

own social circles. The way in which the representativeness of the sample to the demographics of the larger population will be examined is mentioned, but it is unclear how these data will be used. While some reviewers thought that it would be preferable to use recruitment strategies that are designed to lower participation bias rather than after the fact simply assessing whether there is biased participation, other reviewers were not as concerned about the recruitment strategies and thought they seemed reasonable as proposed. In addition, the team has experience and success in using the proposed strategies previously. The application acknowledges that there are potential contamination effects in that some participants likely will have participated in the previous FHF intervention trial. This seems like an important confound, the effects of which cannot be mitigated by simply using statistical covariation. However, the intent in the CRT design to stratify on prior intervention arms may help with this weakness. The rationale for the age range chosen is not clear. In order for the project to be truly preventive and to avoid overlap with non-naïve participants who were not in the previous FHF intervention, it seems that targeting a younger group of participants would have been more ideal. The mono-method and mono-informant reliance on youth self-report regarding violence perpetration is a limitation. The plan to address this by collecting neighborhood-level data on community violence will not help to gauge the effects of the intervention on the individual youth who participates. Perhaps consideration could be given to alternatives such as obtaining caregiver reports or enlisting a friend or peer to provide reports. The long list of self-report measures adds up to a substantial burden on these adolescents, many of whom are likely to not be good readers. Some of the measures listed have brief versions that would be a better alternative, and use of computer-assisted survey delivery that allows participants to have items read to them aloud might be a better strategy as well. The power analysis for Aim 1 is powered to detect an effect size of 0.39. This is a moderate effect size and the odds of that occurring with this intervention seem very low. There is not a power analysis for Aim 2 for the moderation and mediation effects. In terms of the use of LCA, it is not clear that a sufficiently good case is made for how this is a strength above and beyond looking at individual moderating effects. It seems that examining individual moderating effects would be more practical and translatable to being able to target prevention for specific types of youth and specific types of settings. Non-English language speakers will be excluded from the proposed study, which raises a concern about how that would impact generalizability in the particular neighborhoods of the study, especially in DC and the DMV where there may be a considerable number of non-English speakers.

Protection of human subjects is acceptable. Inclusion is acceptable in terms of gender, minority groups, and children. Animal welfare, biohazards, and dual use research are not applicable. An acceptable resource sharing/data management plan (DMP) is provided. In terms of the budget, the percent of efforts seems low for some of the team members (e.g., as low as 5%). Overall, this is an excellent application that is very strong with only some minor weaknesses.

DESCRIPTION (provided by applicant): In response to RFA-CE-22-005, this proposal addresses Objective One: Effectiveness research to evaluate innovative approaches to reducing community violence and racial/ethnic inequities in risk for community violence. Racially and ethnically diverse youth and youth residing in neighborhoods with concentrated disadvantage disproportionately bear the burden of violence. Drawing upon a robust body of scholarship and programming (especially in international settings), and applying an intersectional lens, youth violence can be understood in the context of gender inequities (e.g., adherence to rigid masculinity norms), economic inequities (e.g., unemployment), and race inequities (e.g., racism and discrimination). Chronic, repeated exposure to early trauma and adversity (including violence), persistent experiences of bias-based discrimination, and lack of economic opportunities can impede children’s healthy development and increase the likelihood of both violence victimization and perpetration. Yet few community-driven approaches to violence in the US seek to simultaneously intervene on gender socialization and unequal power dynamics. Programming that explicitly takes into account these intersections and supports youth from diverse backgrounds to address gender-based and structural inequities is both theoretically and empirically novel. The youth empowerment intervention to be refined and evaluated, Forging Hopeful

Futures, will combine economic justice content from job readiness training, racial and gender justice content from gender-transformative programming, and leadership building as a novel multi-level violence prevention intervention. Near program conclusion, youth will be connected with employment opportunities and encouraged to continue participation in social change efforts, with scaffolding offered through community organizations and mentors in each neighborhood. We will conduct a community-partnered cluster randomized trial in 16 neighborhoods impacted by structural inequities and high levels of community violence in Pittsburgh, PA and urban Washington D.C. and Maryland metro areas. Aim 1 will evaluate effectiveness of Forging Hopeful Futures (intervention) compared to wellness check-ins (control) among 14- to 19-year-old youth to decrease self-reported use of physical fighting and threat/injury with a weapon (primary outcome) and multiple related forms of violence (i.e., physical, sexual, and emotional relationship abuse, sexual violence, bullying). Aim 2 will examine mechanisms through which the intervention creates impact and how pre-intervention risk and protective factors moderate intervention effects on multiple forms of violence. Guided by the RE-AIM framework, Aim 3 will examine implementation processes that facilitate intervention acceptability, reach, integration, fidelity, and engagement as well as costs associated with intervention implementation across neighborhoods. Results expected to emerge from this rigorous effectiveness and implementation evaluation will expand our repertoire of effective interventions to address racial, and gender, and economic inequities and reduce youth violence.

PUBLIC HEALTH RELEVANCE: Violence alters the life course of far too many adolescents and disproportionately impacts minoritized youth in neighborhoods burdened by structural inequities and high rates of community violence. Through a community- partnered cluster-randomized trial, we propose to refine and evaluate Forging Hopeful Futures, a youth empowerment intervention that combines economic justice content from job readiness training, racial and gender justice content from gender-transformative programming, and leadership building as a novel multi-level violence prevention intervention. Results expected to emerge from this rigorous effectiveness and implementation evaluation will expand our repertoire of effective interventions to address racial, and gender, and economic inequities and reduce youth violence.

CRITIQUE 1

Significance:	2
Investigator(s):	1
Innovation:	1
Approach:	5
Environment:	1

Overall Impact
Strengths

- This project clearly addresses Objective One by proposing to implement and evaluate an intervention designed to directly target racial and ethnic inequities in risk for community violence.
- The range of forms of violence addressed by the intervention, including gender-based violence, homophobic teasing, bullying, sexual harassment, and sexual violence and coercion, also promises to substantially increase its impact on the participants and the communities in which it will be implemented.
- Not only is the intervention designed to impact the participants and their communities, but the research design itself has the potential to be highly impactful. For example, the workforce development opportunities built into the research participation has the potential to be transformative for the participants.

Weaknesses

- Although data will be gathered on levels of community violence as an outcome variable, there was an absence of attention to social, community, and neighborhood-level variables that might moderate or mediate the impact of the intervention

1. Significance:
Strengths
- This application addresses an important problem and is poised to make a significant impact on the field by attending to the intersectionality of racial, gender, and economic disparities as risk factors for youth violence.
- The results of this project have high potential for generating information related to racial and ethnic inequities, given that the intervention directly targets communities of color and addresses risk factors for violence among youth of color.
- The range of forms of violence addressed by the intervention, including gender-based violence, homophobic teasing, bullying, sexual harassment, and sexual violence and coercion, also promises to substantially increase its impact on the participants and the communities in which it will be implemented.
- In both the short and long term, the project is designed to meet the needs of the communities in which it will be conducted. For example, the workforce development opportunities built into the intervention and the research participation process have the potential to directly address economic inequities and to be transformative for the participants.
- The application is an excellent fit to the NOFO given that it is focused on prevention and addresses factors that can reduce youth violence before it occurs.

Weaknesses
- This reviewer could not find a clear statement regarding which of the NOFO objectives this project addresses, but it is a good fit for Objective 1 given that the focus is on individual factors (youth attitudes) rather than on social or structural community conditions.

2. Investigator(s):
Strengths
- The investigator team is comprised of highly accomplished scholars who have expertise and considerable experience in conducting intervention research with the communities that will be the focus of this project.
- The investigators have laid considerable groundwork for this project, including conducting previous research including many of the facets of the intervention to be tested here.
- The investigators have established strong collaborative relationships with community partners necessary to the community empowerment and participatory action perspectives that inform their approach to this research.

Weaknesses
- No weaknesses are noted.

3. Innovation:
Strengths
- The intervention is highly innovative and novel, addressing the intersectionality of race/ethnicity, gender, and economic inequities in a way that is unique and drawn from a clearly articulated conceptual model.
- The community participatory and empowerment aspects of the methodology also are highly innovative, including engaging a Youth Advisory Committee and involving community partners in the design of the intervention and surveys.
- The examination of youth profiles through LCA promises to make an important contribution to our understanding of intersectionality of risk factors for youth violence and could help to inform future precision medicine approaches that increase the effectiveness of interventions by tailoring and individualizing treatment targets.

- The results have potentially valuable implications for informing future community violence prevention efforts.
- The focus on delivering the intervention outside of school settings is innovative and well-reasoned given that many at-risk youth are not in regular attendance at school.

Weaknesses

- There are missed opportunities to take an innovative holistic social-ecological approach by examining potential community and social level mechanisms of change associated with the intervention.

4. Approach:

Strengths

- The conceptual model underlying the research questions is clear and well-articulated.
- The outcome measures are clearly identified and well operationalized.
- The data to be gathered are appropriate to the research questions and there are no identifiable barriers to the investigator's access to them.
- The data analytic plan is appropriate, methodologically rigorous, and well-articulated.
- The plan includes not only evaluating outcomes, but also testing the underlying conceptual model by examining purported mechanisms of the treatment's effects.
- The design of the control group provides a minimalist form of intervention (wellness check-ins) and is well considered and appropriate given that wait-list control groups are not well received by under resourced communities and their use could reduce motivation and willingness to participate.
- Careful attention is paid to sound implementation science, including ongoing assessment of fidelity through multiple measures, including checklists, interviews, and RA coding of observed sessions.

Weaknesses

- The participant recruitment strategies were one of the least well explicated parts of the research design. Two methods are mentioned: referrals from community agencies and snowball recruitment of youth by other youth. Regarding the first strategy, it appears that community agencies might direct to the intervention youth who already have been involved in violence, which is inconsistent with the focus on prevention in the NOFO. The second strategy seems likely to result in a convenience sample with a limited range of characteristics, given that youth are likely to recruit youth in their own social circles who will be much similar to themselves. Although how the investigators will examine the representativeness of the sample to the demographics of larger population from which it is drawn, it was unclear how they will use those data; preferable would be the implementation of recruitment strategies designed to lower potential participation bias.
- The investigators acknowledge potential "contamination" effects in that some participants are likely to have participated in their previous intervention trial. This seemed like an important confound whose effects cannot be mitigated via the use of statistical covariation.
- Related to the above point, the rationale for the choice of age range of participants was not clear. In order for the project to be truly preventative, and to avoid overlap with non-naïve participants who have participated in their previous intervention trials, targeting a younger group of participants would seem preferable.
- A minor weakness is that as the investigators note, the mono-method, mono-informant reliance on youth self-report regarding violence perpetration is a limitation; their plan to assess neighborhood-level data related to community violence unfortunately will not help to gauge the effects of the intervention on the youth who participate, although it may act as a moderator of the intervention's effectiveness. Alternatives to consider might be to obtain caregiver reports or, as many studies of adolescents do, to enlist a friend to provide reports from an alternative perspective.

- The list of self-report measures adds up to a substantial burden on participants, many of whom are likely to be poor readers. Some of the measures listed have brief versions that might be worth considering. Use of an Audio Computer Assisted Self-Interviewing survey administration system that allows participants to have items read aloud to them might be advantageous.

5. Environment:
Strengths
- The application clearly describes the necessary environmental and institutional supports.
- There are strong existing partnerships among the investigators, the applicant institution, and the community agencies that are key to accessing participants and carrying out the intervention.
- The roles and responsibilities of each member of the research team are clearly described in ways that give confidence in the likelihood that the proposed research will be carried out within the project period.

Weaknesses
- No weaknesses are noted.

Protections for Human Subjects:
Acceptable. Human Subjects involved, no concern
- IRB approval is in progress and the investigators will have a Certificate of Confidentiality.

Data and Safety Monitoring Plan (Applicable for Clinical Trials Only):
Not Applicable

Inclusion of Women, Minorites, and Age (Applicable Only for Human Subjects research and not IRB Exemption #4.):
G1A- Includes both genders, acceptable
M1A- Includes minorities & non-minorities, acceptable
C6A- Includes children & adults (no older adults), acceptable
- Participants in the intervention will be adolescents, intervention facilitators, and community partners who will complete fidelity/feedback ratings and participate in interviews.

Vertebrate Animals:
Not Applicable

Biohazards:
Not Applicable

Dual Use Research of Concern:
Not Applicable

Budget and Period of Support:
Recommend as Requested

Resource Sharing Plans:
Acceptable

Data Management Plan (DMP):
Acceptable

CRITIQUE 2

Significance:	1
Investigator(s):	1

Innovation:	2
Approach:	2
Environment:	1

Overall Impact
Strengths
- The significance and innovation are clearly outlined, and the approach is strong. Several limitations of the approach are noted, and mitigating factors are well described. The investigators have the necessary experience and relationships to carry out this community-based project. The environment is strong, with clear routes for dissemination and translation of findings.

Weaknesses
- Most of the weaknesses listed are minor. The power analysis is conducted to detect an effect of 0.39, but there is no note for how this effect compares to prior effect sizes with similarly conducted intervention trials. This is a moderate effect, which raises the question of whether the study is underpowered to detect effects. There is no power analysis for the moderation and mediation aims.
- Generalizability may be limited by excluding non-English language speakers.
- The link between the program content and the theoretical framework is not clearly laid out.

1. Significance:
Strengths
- The proposed project is responsive to Objective #1 from the NOFO.
- The proposal provides strong background for the significance of gender inequities, racial/ethnic inequities, and economic inequities. There is also a strong link between gender inequities, racial/ethnic inequities, and economic inequities and the outcomes of interest.
- The proposal provides a strong significance for youth empowerment at the developmental time period proposed (age 14-19 Pittsburgh and DC/DMV).

Weaknesses
- PVEST is mentioned at the bottom of the significance section but was not explained or strongly linked to the conceptual framework. The expansion of PVEST as a theoretical framework for implementation of this intervention with minority youth would help to strengthen the significance of this study.

2. Investigator(s):
Strengths
- The project personnel have the experience necessary to carry out the proposed project. They have led many federally funded grants as PIs and Co-Is, including prior cluster randomized trials. The project personnel in Pittsburgh have prior experience collaborating, and the personnel at the Population Research Council have experience collaborating with one another. Additionally, the project personnel have extensive experience in community settings.
- The Population Council has decades of expertise in developing innovative methods to measure gender norms (e.g., GEM scale), and designing, testing, and adapting gender-transformative programs in international settings to promote equitable gender norms, reduce sexual and gender-based violence, address intersections between gender-based violence and community violence, and improve health outcomes. There has also been some translation to US settings (Manhood 2.0).

Weaknesses
- No weaknesses are noted.

3. Innovation:
Strengths

- The community driven approach aiming to intervene in the intersection of gender inequities, racial/ethnic inequities, and economic inequities during this developmental time period is novel.
- Three main innovations are clearly noted: a) conceptual innovation applying an intersectional lens (including the role of gender) to community violence; b) programmatic innovation using novel strategies in gender transformative, intersectional programming and centering racial and gender equity and economic justice as core elements of violence prevention; c) integration of innovative methodological approaches to capture key mediators, examine risk and protective factor profiles, and differential intervention effects across multiple forms of violence.
- Community-level and gender-transformational interventions have been applied extensively in international (low and middle income) settings, yet these approaches and lessons learned have generally not been implemented in US settings.
- Novel measures are used and will be adapted to US settings.
- The expansion to the DMV represents a strength in terms of generalizability of findings.
- The inclusion of the youth advisory council is a strength.

Weaknesses

- The proposal notes the use of LCA as an innovation, but then points to a study that uses the same approach.

4. Approach:
Strengths

- The proposal builds on “The Pittsburgh Study,” a large, county-wide community-partnered collective impact initiative. Participation in the proposed project will be supported by the existing partnership infrastructure.
- The inclusion of multiple sites, including Pittsburgh, Washington, DC, and the DMV metro area is a strength for the generalizability of findings. The CRT stratifies by site and prior intervention arm.
- The curriculum content is clearly laid out.
- The control group is clearly described and well defined.

Weaknesses

- While the theoretical framework is clearly laid out and the link to the outcomes is clear, the link between the program content and the theoretical framework is less clear. For example, Table 2 introduces a number of terms and concepts that are not clearly outlined in the proposal. It is not clear how those concepts relate to the theory of change.
- Curricular content introduces “strengths-based” and “healing centered” approaches, but these were not described in detail above.
- The power analysis for Aim 1 is conducted to detect an effect size of 0.39, but it is not clear how this compares to other effect sizes for similar intervention trials. There isn’t a power analysis for Aim 2 – moderation and mediation.
- The proposal uses LCA for the aim on moderation but does not clearly outline why LCA is a strength above looking at individual factors that moderate outcomes. It seems that looking at individual moderating effects would help with targeting prevention in the future.
- Contamination effects are a concern because participants may have been involved in past interventions from this group. It is not clear if stratification by prior intervention arm addresses this concern.
- Challenges are outlined and addressed clearly, but there are unaddressed questions about generalizability. The exclusion of non-English speakers may limit generalizability for the neighborhoods to the degree that this population is prevalent in the neighborhoods included.

5. Environment:
Strengths

- The environment at the University of Pittsburgh is strong, with multiple Centers and Institutes available to support this project. Additionally, strong dissemination and clinical translation is noted.
- Community partnerships in Pittsburgh are strong through TPS and the proposal aims to build upon the Population Council's emerging partnerships in the DMV.
- A clear management plan with % efforts and working relationships is outlined to carry out the research strategy.

Weaknesses

- No weaknesses are noted.

Protections for Human Subjects:
Acceptable. Human Subjects involved, no concern

Data and Safety Monitoring Plan (Applicable for Clinical Trials Only):
Not Applicable

Inclusion of Women, Minorites, and Age (Applicable Only for Human Subjects research and not IRB Exemption #4.):
G1A- Includes both genders, acceptable
M1A- Includes minorities & non-minorities, acceptable
C6A- Includes children & adults (no older adults), acceptable

Vertebrate Animals:
Not Applicable

Biohazards:
Not Applicable

Dual Use Research of Concern:
Not Applicable

Budget and Period of Support:
Recommended budget modifications or possible overlap identified:

- The budget narrative for Population Council is only detailed for year 1. Clarification for years 2 and 3 would be helpful.

Resource Sharing Plans:
Acceptable

Data Management Plan (DMP):
Acceptable

CRITIQUE 3

Significance:	4
Investigator(s):	3
Innovation:	3
Approach:	2
Environment:	1

Overall Impact
Strengths

- The proposed project takes an innovative approach to community-based youth violence prevention by integrating an economic, racial, and gender equity lens and incorporating youth empowerment. Successful completion of the project will provide important information about an innovative strategy for youth violence prevention, as well as moderators of effectiveness and mechanisms of change.
- The investigators have a range of expertise and are highly qualified to carry out the proposed work, including expertise in youth violence prevention, evaluation of clinical trials, and gender-based violence. They are experienced in administrating and disseminating large-scale research.
- The proposed project integrates implementation and dissemination science and prioritizes sustainability and scalability in the context of robust community partnerships.

Weaknesses

- The project generally takes an individual-level approach to change, even with a racial, gender, and economic equity lens. Community-level change is not targeted and is viewed as exploratory. It is assessed but with insufficient power to detect small effects. This is a moderate weakness.
- The investigator team's level of effort in general seems low to accomplish project goals.

1. Significance:

Strengths

- The proposed program is strongly based in theoretical understandings of youth violence from a structural and cultural perspective as well as existing empirical research on predictors of youth violence. It integrates emerging global research on innovative strategies to reduce violence through promoting economic, racial, and gender equity and justice.
- Successful completion of the project will provide important information about novel community-based strategies that can reduce youth violence, including racial and gender disparities in violence, and that is feasible and scalable.

Weaknesses

- It is not clear how this youth-focused intervention would conceptually lead to changes in community levels of violence, and it appears that individual-level change rather than community-level change is the primary intended outcome.
- The failure to address structural racism and rather provide an inoculation approach or individual-level resilience (e.g., job skills; networking for youth) for mitigating the impact of racism is limiting. In general, the proposed project takes an individual-level approach over a systems-level approach in the intervention strategies.

2. Investigator(s):

Strengths

- The investigator team has a range of expertise that will promote successful completion of the project, including adolescent medicine, public health and epidemiology, biostatistics, and anthropology. The research team also has experience working together; Co-I Abebe is a mentor on Culyba's K23 and has worked with Co-I Miller on multiple sexual violence prevention trials. Co-I Miller has worked with Co-I Pulerwitz on multiple projects.
- The team has strong experience in violence prevention and evaluation research. PI Culyba has worked in the area of youth violence for several years, including in implementation and evaluation of youth violence prevention strategies. She currently has a K award in this area from the National Institute of Child Health and Human Development. Her research has resulted in over 50 publications in this area, and it includes innovative research on the role of racism, adult support, and physical environmental factors. Co-I Abebe has specific expertise in design and evaluation of clinical trials, including leading the statistics components of two randomized trials on youth violence prevention. He is the PI of two, large NIH-funded consortiums. Co-Is Miller and Pulerwitz have extensive research and expertise on addressing gender-based violence, with extensive publications in this area.

Weaknesses
- The level of effort (lows of 5% for Co-Is Miller, Ettinger, Abebe; 10% for Co-Is Pulerwitz and Gottert) seems insufficient to successfully complete the project.
- None of the investigators appear to have experience or expertise in mitigating youths' responses to racism/discrimination or reducing structural racism.

3. Innovation:
Strengths
- The study applies global research on gender transformative programming to work in the US and addresses economic, racial, and gender equity (including equity for LGBTQ+ youth).
- The study explores differential effects across forms of violence and risk and protective factor profiles. It also allows for the testing of mediation to identify mechanisms of change which can be used to refine future implementations.
- The study integrates end-user priorities from the beginning, including incorporating community-identified drivers of violence, using train-the-trainer models with existing partners, providing financial support to community partners to engage in project activities, and supporting the career development of both youth and adults involved in the project.

Weaknesses
- Given the limited research how to effectively promote racial/ethnic identity in a way that buffers against the impact of racism, the risk of unintentional iatrogenic effects seems high. The lack of pilot work on this component contributes to this concern.

4. Approach:
Strengths
- The study is well thought out in terms of maintaining engagement (i.e., incentives to youth for attending sessions; increasing incentives for completing surveys; planning to request a waiver of parent/guardian active consent).
- The analyses are strong in terms of both reducing bias and delving into intervention effects. There are plans for both intent-to-treat analyses, treatment-as-intended analyses, and attrition analyses. The time-series analyses are well suited for the analysis of the community-level archival data. There are plans for examining the extent to which the impact of the intervention varies by baseline involvement in violence as well as demographic characteristics of youth.
- Sources of information include youth self-report and archival data (police records and 911 calls) as well as qualitative interviews and field notes. The mixed methods approach for implementation evaluation is strong.
- In addition to evaluating effectiveness and implementation, the proposed study will also conduct a cost analysis.

Weaknesses
- It appears that no victimization experiences are assessed. It is unclear why this choice was made and limits the ability to fully evaluate the impact of the intervention.
- The power to detect effects is small; this is especially concerning for assessing community-level change, which is likely to be minimal.

5. Environment:
Strengths
- University of Pittsburgh is an R1 university that fosters research and has adequate support for the proposed project. Relevant resources include the Clinical and Translational Science Institute (which has a Community Engagement and Research core), the Center for Research on Health Care Data Center, and an ongoing youth research advisory board. Co-I Miller is the director of the Division of Adolescent and Young Adult Medicine and an investigator on The Pittsburgh Study, which has long-standing relationships with hundreds of community partner organizations across the city.

- In her role as the director of the Empowering Teens to Thrive hospital-based violence prevention program, PI Culyba has ongoing, strong partnerships with community agencies, including the Neighborhood Resilience Project, and institutions such as the Allegheny County Office of Violence Prevention. There are existing data use agreements for accessing police records and call logs.
- The Population Council is a global nonprofit that supports public health research in 50 countries.
- The intervention will take place in the Pittsburgh and Washington, DC, areas, both of which are experiencing increased rates of gun violence and high racial disparities in both violence and poverty.

Weaknesses

- Black youth are a small percentage of the population in Allegheny County, but this is balanced by a higher proportion of Black youth in the DC area and the high disparities experienced by Black youth in Allegheny County.

Protections for Human Subjects:
Acceptable. Human Subjects involved, no concern

- Youth will give informed assent; waiver of parent/guardian active assent will be requested to increase equity in access to services.

Data and Safety Monitoring Plan (Applicable for Clinical Trials Only):
Not Applicable

Inclusion of Women, Minorites, and Age (Applicable Only for Human Subjects research and not IRB Exemption #4.):
G1A- Includes both genders, acceptable
M1A- Includes minorities & non-minorities, acceptable
C6A- Includes children & adults (no older adults), acceptable

- Implementers participating in interviews should also be identified as human subjects.

Vertebrate Animals:
Not Applicable

Biohazards:
Not Applicable

Dual Use Research of Concern:
Not Applicable

Budget and Period of Support:
Recommended budget modifications or possible overlap identified:

- The investigator time and research assistant support (particularly for fidelity monitoring of all sessions) seem insufficient to complete the project.

Resource Sharing Plans:
Acceptable

Data Management Plan (DMP):
Acceptable

THE FOLLOWING SECTIONS WERE PREPARED BY THE SCIENTIFIC REVIEW OFFICER TO SUMMARIZE THE OUTCOME OF DISCUSSIONS OF THE REVIEW COMMITTEE, OR REVIEWERS' WRITTEN CRITIQUES, ON THE FOLLOWING ISSUES:

PROTECTION OF HUMAN SUBJECTS: ACCEPTABLE

INCLUSION OF WOMEN PLAN: ACCEPTABLE
Includes both women and men, which is scientifically acceptable.

INCLUSION OF MINORITIES PLAN: ACCEPTABLE
Includes both minorities and non-minorities, which is scientifically acceptable.

INCLUSION ACROSS THE LIFESPAN: ACCEPTABLE
Includes children and adults (no older adults), which is scientifically acceptable.

COMMITTEE BUDGET RECOMMENDATIONS: Budget modifications are recommended.

- The budget narrative for Population Council is only detailed for year 1. Clarification for years 2 and 3 would be helpful.
- The investigator time and research assistant support (particularly for fidelity monitoring of all sessions) seem insufficient to complete the project.

COMMITTEE RESOURCE SHARING PLAN RECOMMENDATIONS:
The Resource Sharing Plan is acceptable.

COMMITTEE DATA MANAGEMENT PLAN RECOMMENDATIONS:
The Data Management Plan is acceptable.

MEETING ROSTER

National Center for Injury Prevention Special Emphasis Panel
NATIONAL CENTER FOR INJURY PREVENTION AND CONTROL
CE22-005: Research Grants for Preventing Violence and Violence Related Injury (R01)

ZCE1 AW (4B)
05/10/2022

CHAIRPERSON(S)

XIANG, HENRY, MD, PHD, MPH, MBA
PROFESSOR OF MEDICINE
DIRECTOR OF CTR FOR PEDIATRIC TRAUMA RESEARCH
CENTER FOR INJURY RESEARCH AND POLICY
ABIGAIL WEXNER RESEARCH INSTITUTE
AT NATIONWIDE CHILDREN'S HOSPITAL
COLUMBUS, OH 43205

MEMBERS

HENNEBERGER, ANGELA KAY, PHD
RESEARCH ASSISTANT PROFESSOR
UNIVERSITY OF MARYLAND
SCHOOL OF SOCIAL WORK
BALTIMORE, MD 16601

KERIG, PATRICIA K., PHD
PROFESSOR
UNIVERSITY OF UTAH
DEPARTMENT OF PSYCHOLOGY
SALT LAKE CITY, UT 84112

MEHARI, KRISTA RUTH, PHD
ASSISTANT PROFESSOR
DEPARTMENT OF PSYCHOLOGY
COLLEGE OF ARTS AND SCIENCES
UNIVERSITY OF SOUTH ALABAMA
MOBILE, AL 36688

OHMER, MARY L, MPIA, PHD, MSW
ASSOCIATE PROFESSOR, CHAIR, COSA SPECIALIZATION
UNIVERSITY OF PITTSBURGH
SCHOOL OF SOCIAL WORK
PITTSBURGH, PA 15218

SCIENTIFIC REVIEW OFFICER

WILKES, AISHA L, MPH
SCIENTIFIC REVIEW OFFICIAL
NATIONAL CENTER FOR INJURY PREVENTION & CONTROL
NATIONAL CENTER FOR ENVIRONMENTAL HEALTH/ATSDR
EXTRAMURAL RESEARCH PROGRAM OPERATIONS
CENTERS FOR DISEASE CONTROL & PREVENTION
ATLANTA, GA 30341

EXTRAMURAL SUPPORT ASSISTANT

JACKSON, LATANYA D
GRANTS TECHNICAL ASSISTANT
NATIONAL CENTER FOR INJURY PREVENTION & CONTROL
NATIONAL CENTER FOR ENVIRONMENTAL HEALTH/ATSDR
EXTRAMURAL RESEARCH PROGRAM OPERATIONS
CENTERS FOR DISEASE CONTROL & PREVENTION
ATLANTA, GA 30341

OBSERVER

WALTERS, MIKEL, MA, PHD
ACTING DIRECTOR, EXTRAMURAL RESEARCH PROGRAM OPERATIONS
NATIONAL CENTER FOR INJURY PREVENTION & CONTROL
NATIONAL CENTER FOR ENVIRONMENTAL HEALTH/ATSDR
CENTERS FOR DISEASE CONTROL AND PREVENTION
ATLANTA, GA 30341

OTHER REVIEW STAFF

SACCO, JAMES L., MSW
ASSISTANT SCIENTIFIC REVIEW OFFICIAL - ONPAR PRODUCTIONS
NATIONAL CENTER FOR INJURY PREVENTION & CONROL
NATIONAL CENTER FOR ENVIRONMENTAL HEALTH/ATSDR
EXTRAMURAL RESEARCH PROGRAM OPERATIONS
CENTERS FOR DISEASE CONTROL & PREVENTION
ATLANTA, GA 30341

Consultants are required to absent themselves from the room during the review of any application if their presence would constitute or appear to constitute a conflict of interest.
